# Non-Desmoglein Antibodies in Patients With Pemphigus Vulgaris

**DOI:** 10.3389/fimmu.2018.01190

**Published:** 2018-06-04

**Authors:** Kyle T. Amber, Manuel Valdebran, Sergei A. Grando

**Affiliations:** ^1^Department of Dermatology, University of California Irvine, Irvine, CA, United States; ^2^Department of Dermatology, Institute for Immunology, University of California Irvine, Irvine, CA, United States; ^3^Department of Biological Chemistry, Institute for Immunology, University of California Irvine, Irvine, CA, United States

**Keywords:** pemphigus, autoantibodies, secretory pathway Ca^2+^ ATPase, ATP2C1, acantholysis, keratinocyte, acetylcholine

## Abstract

Pemphigus vulgaris (PV) is a potentially life-threatening mucocutaneous autoimmune blistering disease. Patients develop non-healing erosions and blisters due to cell–cell detachment of keratinocytes (acantholysis), with subsequent suprabasal intraepidermal splitting. Identified almost 30 years ago, desmoglein-3 (Dsg3), a Ca^2+^-dependent cell adhesion molecule belonging to the cadherin family, has been considered the “primary” autoantigen in PV. Proteomic studies have identified numerous autoantibodies in patients with PV that have known roles in the physiology and cell adhesion of keratinocytes. Antibodies to these autoantibodies include desmocollins 1 and 3, several muscarinic and nicotinic acetylcholine receptor subtypes, mitochondrial proteins, human leukocyte antigen molecules, thyroid peroxidase, and hSPCA1—the Ca^2+^/Mn^2+^-ATPase encoded by ATP2C1, which is mutated in Hailey–Hailey disease. Several studies have identified direct pathogenic roles of these proteins, or synergistic roles when combined with Dsg3. We review the role of these direct and indirect mechanisms of non-desmoglein autoantibodies in the pathogenesis of PV.

## Introduction

Pemphigus vulgaris (PV) is a potentially life-threatening mucocutaneous autoimmune blistering disease. Patients develop non-healing erosions and blisters due to cell–cell detachment of keratinocytes (acantholysis), with subsequent suprabasal intraepidermal splitting. Identified more than 25 years ago, desmoglein-3 (Dsg3), a Ca^2+^-dependent cell adhesion molecule belonging to the cadherin family, has been considered the “primary” autoantigen in PV.

While convincing evidence has supported the pathogenic role of anti-Dsg antibody-mediated acantholysis, there has been a shift in our understanding of the disease from steric hindrance by autoantibodies to modification of cell metabolism and signaling, and structural alterations in the desmosome affecting cell adhesion (1–3).

Proteomic studies have identified numerous autoantibodies in patients with PV that have known roles in the physiology and cell adhesion of keratinocytes. These autoantibodies include desmocollins (Dsc) 1 and 3, several muscarinic and nicotinic acetylcholine receptor (nAChR) subtypes, mitochondrial proteins, human leukocyte antigen (HLA) molecules, thyroid peroxidase (TPO), and hSPCA1—the Ca^2+^/Mn^2+^-ATPase encoded by ATP2C1, which is mutated in Hailey–Hailey disease (HHD).

The presence of numerous potentially pathogenic autoantibodies in pemphigus points toward a need to understand these non-Dsg antibodies. While several of the most prevalent non-Dsg autoantibodies have been characterized regarding their effect on keratinocyte biology, a majority of identified autoantibodies remain poorly understood. We review the direct and indirect pathogenic mechanisms of non-Dsg autoantibodies in PV.

## Major Defined Autoantigens

### Desmocollins

Desmocollins (Dsc) and Dsgs are two specialized Ca^2+^-dependent cadherin subfamilies that provide structure to the desmosomes (4). On the cell surface, Dsc and Dsg bind to each other, providing support, and meditating connections between intermediate filaments of neighboring cells (5, 6). In addition, this cadherin complex forms an anchor for keratin intermediate filaments which attach to the inner cytoplasmic surface (6).

Desmocollins play an important role in cell-to-cell adhesion. This role was demonstrated by Spindler et al. (7) through the application of monoclonal antibodies against extracellular domains of Dsc3 in human skin model which resulted in intraepidermal blister formation. They also provided evidence of homophilic and heterophilic trans-interaction of Dsc3 with Dsg1. Their data showed that Dsg-1 IgG antibodies reduces the adhesion of Dsc-3 to the surface of keratinocytes, possibly by targeting the Dsc3/Dsg1 binding on keratinocyte cell surface (7).

Previously, IgA autoantibodies to Dsc1 were identified in subcorneal pustular dermatosis (8). However, various reports of IgG autoantibodies against the three different Dsc have been found in paraneoplastic pemphigus/paraneoplastic autoimmune multiorgan syndrome (PNP/PAMS), PV, pemphigus foliaceus (PF), pemphigus vegetans (PVeg), and pemphigus herpetiformis (PH) (9).

In a study of a large cohort of pemphigus patients, autoantibodies to Dsc 1 and 3 were present in 44% of patients, with only 7% in matched controls (10). Ishii et al. reported their findings related to the detection of anti-Dsc antibodies in a series of 164 pemphigus cases. Anti-Dsc antibodies were found in 3 of 22 PV cases (13%) and 3 of 18 PF cases (18%). By contrast, 53 of 79 PNP/PAMS cases (67%) demonstrated anti-Dsc 1–3 antibodies. Specifically, Dsc3 antibodies were detected in roughly 60% of the cases, Dsc 2 in 37%, and Dsc 3 in 16.5% of cases. In PH and PVeg, around 40% of sera showed strong reactivity with Dsc1–3 (11).

Dettmann et al. studied serum reactivity with Dsc 1, 2, and 3 serum autoantibodies in various groups of patients with pemphigus. In their first cohort consisting of 102 PV patients with oral lesions with positive direct immunofluorescence (DIF) and the presence of anti-Dsg3 IgG, no antibodies were found against Dsc 1, 2, and 3. The second cohort was composed of 24 patients with oral lesions but no detectable Dsg3 autoantibodies; therein IgG antibodies against Dsc 3 were found in one case. The third cohort composed of 23 patients with PNP/PAMS mostly reactive against anti-Dsg3 and envoplakin, from whom anti-Dsc 2 and anti-Dsc 3 IgG autoantibodies were identified in two patients, respectively (9). A fourth cohort consisting of sera from 749 patients of the International Autoimmune Bullous Diseases Study Group was also analyzed for reactivity with epitopes of Dsc. Of these patients, 333 were diagnosed with pemphigus. Investigators found 14 (4%) Dsc reactive cases; 3 were from patients with PNP/PAMS, 2 from patients with PVeg. 2 of 14 sera were from PV patients in whom no Dsg 1 or 3 reactivity was found. Three reactive cases of PF showed Dsc 1-specific IgG. Of 5 Dsg3-negative sera, 333 (40%) showed reactivity with Dsc 1. Thus, testing for autoantibodies against Dscs was only recommended in patients with atypical pemphigus (9).

### hSPCA1

The ATP2C1 gene codes for a magnesium-dependent enzyme that catalyzes the hydrolysis of Ca^2+^-transport ATP. This secretory pathway Ca^2+^ ATPase pump is expressed in the Golgi apparatus (GA) which is involved in the transport of calcium and manganese ions from the cytosol to the lumen of the GA. Mutations in ATP2C1 results in depletion of Ca^2+^ stores in the GA with increased Ca^2+^ in the cytosol (12). Low Ca^2+^ concentration in the GA could impair posttranslational processing of important desmosomal proteins such as Dsg and Dsc. As a result, acantholysis develops. These mutations have been found in HHD (12, 13).

Clinical and pathological similarities between HHD and PV exist. Clinically, vesicle and bulla formation may be present in early lesions of HHD. However, features of established lesions include eroded scaly plaques in symmetrical and intertriginous distribution, commonly in axillae, groin, neck, inframammary fold, and perineum (14). On the other hand, features of PV include flaccid blisters and erosions on mucosa and sometime also skin. Histologically, in PV, suprabasal acantholysis is present with intraepidermal bulla formation and a tombstone pattern of the remaining cells of the basal layer. Although acantholysis is also present in HHD, it involves all epidermal layers resembling the dilapidated brick wall (14). Invariably, DIF is negative in HHD.

Over 125 pathogenic mutations through the ATP2C1 gene have been described in HHD (15, 16). Approximately 20% are nonsense mutations, 30% are frame-shift mutations with premature termination codons, and 28% are missense mutations (17). Investigators suggest that haploinsufficiency is the mechanism of dominant inheritance observed in this entity (18).

Autoantibodies against hSPCA1 are seen in 43% of patients with PV compared to 8% in matched controls (OR = 8.51) (10). Given the similarities in clinical phenotype and role of cell disadhesion in both of these diseases, it is tempting to suspect that anti-hSPCA1 autoantibodies have an active role in PV. To date, however, there have been no studies assessing the pathogenic role of these autoantibodies.

### Cholinergic Receptors

The human epidermis has an elaborate non-neuronal cholinergic system composed of an axis that involves keratinocyte acetylcholine (ACh) enzymes involved in ACh synthesis and degradation, and nicotinic and muscarinic acetylcholine receptors (AChRs) (19). Keratinocyte ACh plays a role in the regulation of cell–cell and cell–matrix adhesion. This mechanism is accomplished through AChR signaling. These receptors have a regulatory effect by activation or inhibition of kinase cascades. Resulting outcome will lead to either upregulation or downregulation of the expression of cell adhesion molecules such as cadherins and integrins (19).

Muscarinic acetylcholine receptors (mAChRs) are single-subunit transmembrane glycoproteins composed of five receptor subtypes (M_1_–M_5_). Odd-numbered mAChRs bind to pertussis toxin-insensitive G proteins stimulating phospholipase C enzymes that hydrolyze phosphatidylinositol 4,5-bisphosphate, yielding the second messenger molecules inositol 1,4,5-trisphosphate (IP3) and diacyl glycerol which in turn control intracellular levels of Ca^2+^ and protein kinase C activity, respectively. Even-numbered mAChR subtypes couple to pertussis toxin-sensitive G proteins ultimately leading to inhibition of adenyl cyclase, and weakly stimulating phospholipase C; they also rectify K^+^ channels, and augment arachidonic acid release (19). *In vitro* experiments have shown that mAChR activation may prevent, stop, and reverse acantholysis mediated by pemphigus antibodies (20). Autoantibodies against mAChRs have been identified in the serum of 85–100% of pemphigus patients (21, 22). Lakshmi et al. prospectively evaluated the disease severity of 45 patients with pemphigus who were followed up at baseline, 3 months, and 15 months. They collected sera from these patients to assess the titers of antibodies against Dsg1/Dsg3 and anti-M3 mAChR to correlate them with disease severity and response to therapy. They found that antibody titers correlated significantly with disease activity and that anti-M3 mAChR antibodies were present in all cases (22).

Nicotinic acetylcholine receptors are members of the superfamily of ligand-gated ion channel proteins, mediating Na^+^ and Ca^2+^ influx and K^+^ efflux. These receptors are not only present on the surface of keratinocytes but also on the mitochondrial outer membrane (23). Mitochondrial-nAChRs inhibit mitochondrial permeability transition pore (mPTP) opening, restraining cytochrome *c* (CytC) release, thereby preventing apoptosis (24). IgG from patients with PV bind to several mitochondrial-nAChR subtypes (α3, α5, α7, α9, α10, β2, and β4), resulting in swelling of mitochondria, rupture of outer membrane, and release of CytC caused by mPTP opening. Moreover, CytC induces apoptosome formation with activation of caspase-9 with subsequent induction of apoptosis (25).

Pemphaxin (PX) is a 75-kDa annexin also known as annexin 31 or ANXA9 (26) which was discovered by screening of keratinocyte λgt11 cDNA expression library with PVIgG antibodies. PX acts as an AChR with dual muscarinic and nicotinic pharmacology. *In vitro* studies demonstrated that anti-PX antibodies induced acantholysis in keratinocyte monolayers; confirmation was done with immunofluorescence studies showing positivity in a net-like pattern. *In vivo* experiments demonstrated that while adsorption of anti-PX autoantibody abolished acantholytic activity of PV IgG fraction, adding it back to the preabsorbed fraction restored the acantholytic activity of PV IgG fraction, although anti-PX autoantibody alone did not cause clinically evident skin blisters (27). This finding indicates that PV results from synergistic action of several antibodies to different self-antigens, including AChRs (28). Simultaneous and synergistic action of PV IgGs against the cell membrane and mitochondrial-nAChRs inactivates adhesion molecules and opening of mPTP. Affected keratinocytes shrink and detach from neighboring cells. Therein, antibodies to desmosomal components prevent keratinocyte from re-attachment making keratinocyte detachment irreversible (29).

### Anti-Mitochondrial Proteins

Anti-mitochondrial antibodies (AMA) play an important role in the pathogenesis of PV as they can trigger the intrinsic apoptotic pathway. Adsorption of AMA prevents acantholysis (30). Autoantibodies against numerous mitochondrial antigens are seen in patients with PV, as summarized in Table 1. Other studies evaluating AMA in patients with pemphigus included that by Marchenko et al., which found AMA in 100% (6/6) of sera of patients with PV when studying penetration of PVIgG into the subcellular mitochondrial fraction (30). Experiments conducted by Chernyavsky et al. (25) found AMA against different subunits of mitochondrial ACh receptors in 100% (5/5) sera from patients who had anti-nAChR antibodies.

**Table 1:** Mitochondrial autoantibodies in patients with PV with an incidence greater than 5% and OR > 2 when compared with healthy controls (10).

Antigen	Symbol	Incidence in PV (%)	Incidence in controls (%)	OR
Mitochondrial processing peptidase beta subunit	PMPCB	31	4	8.47
Cytochrome b5 outer mitochondrial membrane isoform precursor	CYB5B	19	1	13.07
Carnitine *O*-palmitoyltransferase I, mitochondrial muscle isoform	CPT1B	18	5	3.51
Peptidase (mitochondrial processing) alpha	PMPCA	16	4	3.75
Mitochondrial import inner membrane translocase subunit TIM13 B	TIMM13	16	4	4.50
Carnitine *O*-palmitoyltransferase I, mitochondrial liver isoform	CPT1A	13	4	3.05
Mitochondrial uncoupling protein 2 (UCP20)	UCP20	10	5	2.02
Solute carrier family 25 (mitochondrial carrier; citrate transporter, member 5)	SLC25A5	10	4	2.27
Solute carrier family 25 (mitochondrial carrier; peroxisomal membrane protein) member 17	SLC25A5	8	1	5.75
Mitochondrial intermediate peptidase	MIPEP	7	1	9.93
Mitochondrial import inner membrane translocase subunit	TIMM22	6	1	3.92

Anti-mitochondrial antibodies bind mitochondrial proteins eliciting the opening of the mPMP which cause massive swelling of mitochondria, rupture of outer membrane, release of CytC, and subsequent activation of caspase-9 (31). In so doing, AMA can complement pro-acantholytic actions of other types of non-Dsg antibodies launching a downstream signaling event involving Src, epidermal growth factor receptor kinase, p38 mitogen-activated protein kinase (p38MAPK), and c-Jun N-terminal kinase (30).

Chen et al. (29) demonstrated that PV IgGs including AMA couple with neonatal Fc receptor (FcRn) on the cell membrane. The PV IgGs–FcRn complex allows the entrance of AMA to the keratinocyte. Once in the cytosol, they dissociate and are trafficked to the mitochondria where they trigger proapoptotic events with subsequent CytC release and activation of caspase-9 (25, 30). These events correlate with the shrinkage of basal keratinocytes seen in histologic sections (29).

Upon recovery, new desmosomes are extended toward neighboring keratinocytes; however, anti-Dsg antibodies prevent bonding of new desmosomes due to steric hindrance, thereby acantholysis becomes an irreversible process (29). The term “apoptolysis” has been introduced to denote these apoptotic and acantholytic events (30).

Overexpression of suppression of tumorigenicity 18 gene (ST18) in keratinocytes of predisposed individuals appears to increase susceptibility of cells to apoptosis and immune dysregulation (32–34). Interestingly, ST18 is overexpressed in PV non-lesional skin when compared with the skin of healthy individuals, indicating a possible predisposition to pemphigus (32). A variant in the promoter region drives increased gene transcription in a p53/p63-dependent manner. This polymorphism, however, appears to be most associated with PV arising in Jewish and Egyptian patients, rather than German, or Chinese (32, 35). Sarig et al. did, however, note an increase of ST18 in patients with psoriasis. Thus, it unclear whether ST18 overexpression simply is associated with inflammatory processes.

### Thyroid Peroxide Antibodies

Thyroid peroxidase (TPO), originally described as thyroid microsomal antigen, is a member of the thyroid autoantigens which includes thyroglobulin and thyroid-stimulating hormone receptor (36). TPO is a glycoprotein present on the apical surface of thyroid follicular cells (36); it is involved in the synthesis of T3 and T4, catalyzing several steps in the process (37). Approximately 85–90% of patients with chronic thyroiditis have anti-TPO antibodies (38); therefore, these antibodies are considered to be the hallmark of autoimmune thyroid disease (ATD), particularly, Hashimoto’s thyroiditis, postpartum thyroiditis, and Grave’s disease (37).

Several studies have documented the association between PV and the presence of anti-TPO antibodies as it is summarized in Table 2. The mean percentage of pemphigus patient with anti-TPO antibodies among all these studies was 19% (3.6–40%), which is well above the standard incidence of anti-TPO autoantibodies. Nevertheless, larger prospective multi-centric studies are needed to further characterize this association.

**Table 2:** Current studies on the incidence of thyroid autoantibodies and thyroid disease in patients with pemphigus.

Study	Country	Patients	Incidence
Pitoia et al. (39)	Argentina	PV (*n* = 15)	
		Anti-TPO	6 (40%)
		Hashimoto thyroiditis	1 (6.6%)

Michailidou et al. (40)	Greece	PV (*n* = 129)	
		Incidence of Thyroid disease	2 (2.6%)

Ansar et al. (41)	Iran	PV (*n* = 22)	
		Anti-TPO	5 (22%)

Daneshpazhooh et al. (42)	Iran	PV (*n* = 75)	
		Anti-TPO	12 (16%)

Leshem et al. (43)	Israel	PV and PF (*n* = 110)	
		Anti-TPO	4 (3.6%)

Kavala et al. (44)	Turkey	PV (*n* = 80)	
		Altered thyroid function test and anti-thyroid Ab	13 (16%)
		Anti-TPO	6 (8%)
		Anti-Tg	2 (2.5%)
		Primary thyroid disease (PTD)	13 (16%)
		Hashimoto thyroiditis	7 (9%)

Ameri et al. (45)	Italy	PV (*n* = 25)	
		Anti-TPO	6 (24%)

Parameswaran et al. (46)	USA	PV (*n* = 230)	
		Group database	
		ATD	23 (10%)
		PV (*n* = 171)	
		Online survey	
		ATD	16 (9.36%)
		PV (*n* = 393)	
		IPPF registry	
		ATD	36 (9.16%)

*PV, pemphigus vulgaris; anti-TPO, anti-thyroid peroxidase; anti-Tg, anti-thyroglobulin; TSH, thyroid-stimulating hormone; IPPF, International Pemphigus and Pemphigoid Foundation; ATD, autoimmune thyroid disease*.

The link between thyroid and skin development and homeostasis has been well documented (47–49). This relationship correlates with the ability of thyroid hormones to bind nuclear receptors with consequent effects on proliferation of keratinocytes and fibroblasts (50, 51). However, the role of thyroid proteins in cell-to-cell adhesion remains poorly understood (51).

### Plakophilin 3

Plakophilin 3 is a member of the armadillo family of proteins; this protein has an important role in the generation of a stable desmosome due to its multiple interactions with several proteins such as desmosomal cadherins, Dsg1, Dsg3, desmoplakin, plakoglobins, and epithelial keratin 18 (52, 53).

Autoreactivity against plakophilin-3 (PKP3) has been demonstrated by Lambert et al. in five PNP/PAMS (100%) sera and in one PV (25%) serum in a series that evaluated sera from five PNP, four PV, two PF, five bullous pemphigoid (BP), one cicatricial pemphigoid, and one linear IgA dermatosis (53). In a large study of PV patients using proteomic technique, 43% of PV patients had autoantibodies targeting plakophilin 3, compared with 7% of matched controls (OR = 6.56) (10).

Preliminary experiments done by Sklyarova et al. have demonstrated that mice deficient in PKP3 show alteration and rearrangement of desmosomes in the epidermis and hair follicles (54). While desmosomes and adherens junctions were significantly altered, compensatory changes in junctional proteins were seen, with upregulation of desmoplakin, plakophilin 1 and 2, E-cadherin, and β-catenin. These mice were also prone to dermatitis. Thus, while it appears unlikely that loss of PKP3 can lead to the clinical phenotype in pemphigus, it is one key to a dense network of proteins that stabilize desmosomes.

### E-Cadherin

E-cadherins along with P-cadherin are the classical cadherins forming adherens junctions, which in conjunction with desmosomes mediate cell-to-cell adhesion (55). Overexpression of E-cadherin has been observed as a result of disruption of desmosomes by PV autoantibodies. E-cadherin partially compensates for loss of cell cohesion, rescuing the cell but also attenuating activation of p38MAPK. E-cadherin is required to assemble Dsg3 into desmosomes (55).

Oliveira et al. and Evangelista et al. have demonstrated antibodies targeting E-cadherin in sera of pemphigus patients (56, 57). One study identified autoantibodies in 78% of mucocutaneous pemphigus vulgaris (mcPV) (*n* = 62) and 33% (*n* = 18) of those with mucosal involvement. Moderate correlation was found between the index values of E-cadherin and Dsg1 antigen/antibody, but no correlation with Dsg3; this finding suggested that antibodies against E-cadherin might cross-react with Dsg1 (or *vice versa*) (57). Similar findings were reported by Evangelista et al. who demonstrated anti-E-cadherin antibodies in 100% of sera of patients with pemphigus foliaceus (*n* = 13) and fogo selvagem (*n* = 15); autoantibodies were observed in 79% of those with mcPV and none of the mucosal-type PV (*n* = 7) (56). More recent proteomic studies of patients with PV demonstrate autoantibodies against E-cadherin in 31% of pemphigus patients compared with 7% of healthy controls (OR = 4.29) (10). Thus, while further pathogenicity studies are needed, the already published data suggests an important synergistic role of E-cadherin autoantibodies in the pathogenesis of pemphigus.

### Plakoglobin

Plakoglobin is a member of the Armadillo family of adhesion and signaling proteins. It has a key role in the organization of desmosomes (58, 59). The highly conserved intracytoplasmic–cadherin-like segment of Dsgs is required for direct binding of Dsgs to plakoglobin (60). Silencing of plakoglobin results in p38MAPK-dependent cell disadhesion in cultured keratinocytes. Plakoglobin additionally regulates levels of Dsg3 (61). Interestingly, inhibition of p38MAPK can prevent PV IgG-induced blistering (62). In PV, there is an accumulation of c-Myc, due to a decrease in plakoglobin-mediated suppression of c-Myc (63). Plakoglobin is a principle effector of PV IgG downstream signaling (64–66). Plakoglobin can additionally regulate the promoters of Dsc2 and Dsc3 (67). While a compensatory increase in beta-catenin can be seen in the setting of plakoglobin silencing, it is insufficient for maintaining normal levels of plakophilin-1 or desmoplakin in the desmosomal plaque (68).

Plakoglobin is precipitated from sera of PV and PF patients, linked to Dsgs (69). It is internalized in combination with Dsg3 and PV IgG, resulting in retraction of keratin filaments (70). PV IgG may also promote separation of Dsg3 and plakoglobin (71). This is consistent with immunohistochemical studies in PV, in which plakoglobin staining was displaced toward the nucleus in comparison to healthy control (72).

Larger studies of autoantibodies in patients with PV demonstrated that 26% of patients carried autoantibodies against junctional plakoglobin, compared with 5% of controls (OR = 5.15) (10). Ishii et al. likewise described a patient with PNP/PAMS who had immunoreactivity against plakoglobin (73). Whether these anti-plakoglobin autoantibodies participate in the plakoglobin-Dsg3 dissociation remains to be determined.

### FcεRI

Human immunoglobulin E (IgE) molecules bind with very high affinity to receptors on the surface of basophils and mast cells. As a result, mast cells release histamine, leukotrienes, prostaglandins, and cytokines (74). Basophils and mast cells can be activated by cross-linking of IgE interacting with antigens (direct anaphylaxis) or by antibodies directed against Fcε chain of IgE, or against the epitopes of α chain of FcεRI or anti-IgG acting on IgG–IgE complexes bound to FcεRI (74).

Tissue specific or systemic autoimmunity might lower the threshold of immunocompetent cells to recognize FcεRI. For this reason, Fiebiger et al. (75) in their study on anti-FcεRIα autoantibodies in chronic urticaria, included sera from patients with systemic and skin-specific autoimmune diseases including systemic lupus erythematosus, dermatomyositis, BP, and PV. Interestingly, anti-FcεRIα antibodies were found in all groups. As opposed to chronic urticaria, antibodies from the autoimmune cutaneous diseases group lacked complement-activation properties, potentially limiting the activation of basophils and subsequent histamine release (75). Sera from 28 PV patients were analyzed by ELISA, of which 39% were positive for anti-FCεR1 antibodies. Pronounced IgG reactivity against Western-blotted recombinant soluble FcεRIα was found in sera of 2 PV patients. Particularly, IgG2 and IgG4 subtypes were found in these patients (75).

The significance of the anti-FcεRIα antibodies in pemphigus remains unclear.

### Other Autoantibodies

While the above discussed non-Dsg autoantibodies target known participants in cell disadhesion and autoimmunity, numerous other autoantibodies have been detected in patients with pemphigus (10). A better understanding of these and other non-Dsg antibodies will provide significant insight into the pathogenesis of pemphigus.

## HLA as a Link Between Different Implicated Autoantigens

The HLA region is located on chromosome 6, present only in humans coding for the major histocompatibility complex (MHC) genes. MHC class I genes are known as HLA-A, HLA-B, and HLA-C; coding for proteins present on the surface of almost all cells. MHC class II genes are located within the HLA-D region on chromosome 6 which contains three subregions: DP, DQ, and DR. Each subregion has one expressed α and β chain gene. Six genes are contained: HLA-DPA1, HLA-DPB1, HLA-DQA1, HLA-DQB1, HLA-DRA, and HLA-DRB1 (76).

There is a strong association between PV and HLA class II genes, especially DR4 and DR14. Further analyses on particular allele subtypes have demonstrated an association with the DRB1*0402, DRB1*1401, DRB1*1404, DRB1*1454, DQB1*0503, and DQB1*0302 alleles. In fact, more than 95% of PV patients carry one the following alleles: DRB1*0402 or DQB1*0503 (2, 77, 78). Particularly, these last two alleles are strongly associated with the Ashkenazi Jewish population (79). PV has been associated with HLA class I including those with HLA-A3, A10, A26, B15, B35, B38, B44, and B60; however, their significance is yet to be elucidated (77). Susceptibility to PF has been linked to the presence of DR4, DR14, and DR1, although no single DR4 or DR14 allele was associated with the PF (80).

High-risk HLA alleles can efficiently accommodate autoantigen-derived peptides, thus eliciting a T-cell-mediated response (78). At the same time, B-cells can be activated by anti-desmosomal CD4 T effector cells.

In contrast to antigen presentation, MHC II autoantibodies can be seen in up to 45% of patients with PV compared 7% in healthy controls (OR = 6.22), with autoantibodies against numerous MHC I and MHC II molecules (10). The functional role of these autoantibodies remains unclear.

Sajda et al. studied a panel of different autoantibodies present in sera of 40 PV patients, 20 healthy relatives, and 20 unrelated controls. Among these antibodies, they identified five which were more significantly associated with PV including Dsg3, mAChR3, mAChR4, mAChR5, and TPO. Of particular interest, non-Dsg autoantibodies in sera of healthy relatives of patients with PV were also seen. Subsequently, they performed HLA analysis clustering the patients into those who had presence or absence of PV-associated HLA alleles DRB1*0402 and/or DQB1*0503. Interestingly, most healthy relatives who presented with these alleles had similar antibody profiles to that of active pemphigus patients, whereas relatives who were negative for the risk alleles had antibody profiles similar to those of unrelated controls (81). Further findings revealed that autoantibody levels on HLA+ and HLA− relatives were comparable, thus implying that further genetic and environmental factors may lead to clinical pathology and remains to be studied (81). These data highlight the importance of HLA as a driver for the breakdown of self-tolerance and the specificity of the autoimmune response.

## Conclusion

The pathogenesis of pemphigus is a complex process involving autoantibodies against numerous structural and metabolic proteins that regulate keratinocyte adhesion and survival. Several of these autoantibodies have been confirmed to have a pathogenic role in pemphigus, by altering the desmosomal plaque, synergistically complementing classic anti-Dsg autoantibody action, or altering mitochondrial physiology. In light of the hundreds of autoantibodies present in patients with PV, only few remain characterized. Thus, significant work is needed to determine the pathogenicity of these autoantibodies.

## Author Contributions

Substantial contributions to the conception or design of the work; or the acquisition, analysis, or interpretation of data for the work; drafting the work or revising it critically for important intellectual content; final approval of the version to be published; agreement to be accountable for all aspects of the work in ensuring that questions related to the accuracy or integrity of any part of the work are appropriately investigated and resolved: KA, MV, and SG.

## Conflict of Interest Statement

The authors declare that the research was conducted in the absence of any commercial or financial relationships that could be construed as a potential conflict of interest.

## Abbreviations

ACh, acetylcholine; AChR, acetylcholine receptor; AMA, anti-mitochondrial antibodies; CytC, cytochrome *c*; DIF, direct immunofluorescence; Dsc, desmocollin; Dsg, desmoglein; FcRn, neonatal Fc receptor; HHD, Hailey–Hailey disease; HLA, human leukocyte antigen; mAChR, muscarinic acetylcholine receptor; mcPV, mucocutaneous pemphigus vulgaris; MHC, major histocompatibility complex; mPTP, mitochondrial permeability transition pore; nAChR, nicotinic acetylcholine receptor; PF, pemphigus foliaceus; PH, pemphigus herpetiformis; PKP3, plakophilin-3; PNP/PAMS, paraneoplastic pemphigus/paraneoplastic autoimmune multiorgan syndrome; PV, pemphigus vulgaris*;* PVeg, pemphigus vegetans; PV IgG, IgG from pemphigus vulgaris patients; PX, pemphaxin.
